# How are self-rated health and diagnosed disease related to early or deferred retirement? A cross-sectional study of employees aged 55-64

**DOI:** 10.1186/s12889-016-3438-6

**Published:** 2016-08-26

**Authors:** Kerstin Nilsson, Anna Rignell Hydbom, Lars Rylander

**Affiliations:** 1 Divison of Occupational and Environmental Medicine, Lund University, Box 188, SE-221 85 Lund, Sweden; 2 Swedish University of Agricultural Sciences, Work Sciences, Business Economics and Environmental Psychology, Box 88, SE-230 53 Alnarp, Sweden

**Keywords:** Diagnosed disease, Extended work life, Health, Older workers, Retirement, Self-rated health

## Abstract

**Background:**

More people will probably continue working into old age in the future due to the increased size of aging populations in many countries. We therefore need to know more about older workers’ health in relation to their work situation and retirement. This study is a part of a theoretical development of older workers’ situations. Older workers’ situations are theoretically themed in nine areas by the authors of this study. The aims of the study were to investigate the relationship between: i) diagnosed disease and factors in older workers’ situations, theoretically themed in nine areas; ii) self-rated health and factors in older workers’ situations, theoretically themed in nine areas; iii) diagnosed disease and self-rated health; and iv) the relationships between these health measures and retirement.

**Methods:**

A questionnaire-based cross-sectional study, using logistic regression, with 1,756 health care personnel aged 55–64 years. The questionnaire used gave an overview of most different areas in the older workers’ situations.

**Result:**

There was a difference in the participants’ frequency of objectively specified diagnosed disease and their subjectively experienced self-rated health. A bad self-rated health was related higher to early retirement than diagnosed diseases. In the multivariate model, having ‘Diagnosed disease’ was not significantly related to whether older workers thought they could not work beyond 65 years of age. A bad ‘Self-rated health’ was also more highly related to whether older workers thought they could not work beyond 65 years, than if the respondents stated that a ‘Diagnosed disease is a hindrance in my daily work’ in the multivariate model.

**Conclusion:**

This study showed an important difference between older workers’ own experiences and the effect of their self-rated health and their diagnosed diseases. Subjective self-rated health seems to be more important to people’s retirement planning than diagnosed disease. The most important factors affecting older workers’ self-rated health was the degree to which they felt physically and mentally fatigued, their possibilities for revitalization, and issues of work satisfaction, age discrimination and attitudes of managers to them as seniors.

## Background

The social occupational pension system was introduced to make it economically possible for people to withdraw from working life when their health declined as a result of old age. To extend working life for older workers is nowadays high on the agenda in many countries considering their social pension system, because the old age dependency ratio will increase along with the rising proportion of older people in most industrialised countries [1–5]. Due to that, many countries will have to increase the number of working hours in the economy in order to maintain the welfare state and, will therefore have to motivate people to work into older age [1–4].

Health is described as one of the most important factors affecting disability and retirement age [6–18]. Defining a person’s health is complex and due to its different definitions, health can be rated in different ways. Some of the most commonly used health measures in investigations are self-rated health and diagnosed diseases [27–29]. Diagnosed diseases as determined by a physician is an objective way to rate health, but self-rated health is an individual’s subjective experience of their own health [30]. The social insurance system in many countries uses diagnosis to define a person’s eligibility for sick leave and retirement, although self-rated health has been found to be a better predictor for disability retirement than medical diagnosis in some studies [31–33]. People in their 50s reported a lower level of good self-rated health than their diagnosed diseases, but 10 years later the same persons reported a higher level of good self-rated health as well as a greater amount of diagnosed disease [38]. Some studies have also stated that self-rated health is a better predictor of mortality than more objective health parameters like diagnosed disease phase [34–37].

Retirement is described to be both positive and negative to an individual’s health [19]. Some of those who participate in working life at an older age (65+) perceive themselves to have better mental and physical health due to their meaningful tasks and physical activities in work [11, 20–22]. Others report that mental and physical fatigue decrease after retirement [11, 23, 24]. Some studies have stated that health improves after retirement among those with a poor work environment and who had health complaints before retirement, but this is less apparent among workers experiencing low demands, high satisfaction and a high occupational grade in working life [25, 26]. However, a person’s health status is important to whether they can participate and continue working, and it is their first concern regarding retirement decisions [11]. Regarding the complexity of health definitions, deeper knowledge is required regarding the relationships and differences between diagnosed disease and self-rated health [28, 29, 36, 38]. It is currently unknown whether diagnosed health or self-rated health is the best predictor of retirement.

Different factors of importance to work life participation have been divided into theoretical themes [39, 40] including where those theoretical themes comprise nine areas [41–44]. The overall aim of this study was to explore if diagnosed health or self-rated health was a better predictor of retirement age. The objectives were to investigate the relation between: i) diagnosed disease and factors in older workers’ situations within nine areas of theoretical themes; ii) self-rated health and factors in older workers’ situations within nine areas of theoretical themes; iii) diagnosed disease and self-rated health; and iv) the relationships between these health measures and retirement.

## Methods

### Study population

The study population consisted of 2,822 employees aged 55–64 years and identified in the official employment register for healthcare and medical services in Scania, the most southern county in Sweden. The study design was cross-sectional, and these individuals were sent a questionnaire by post to measure their attitudes and self-stated potential to work an extended working life (see detailed description below). After two reminders, 1,815 individuals responded, which corresponds to a response rate of 64 %. However, 59 individuals who did not answer the questions about their self-rated health and diagnosed disease were excluded from the study, which left 1,756 individuals in the final study population.

The median age among the participants was 58 years, which was similar to that of non-participants (58 years). The majority of the participants who received a questionnaire were women (85 %); this reflects that more women than men work in healthcare and medical services. In addition, the response rate was better among women (68 %) than men (59 %). Among the participants with a partner, 61 % had a partner who worked full-time, 13 % had a partner who worked part-time, and 26 % had a partner who did not work at all.

The distribution of occupations among the participants was: 21 % nurses, 20 % medical secretaries, 17 % physicians, 16 % nursing assistants, 7 % physiotherapists, 7 % welfare officers, 4 % psychologists, 5 % psychiatric nursing assistants, and 3 % occupational therapists. The proportion of non-responders was highest among physicians (25 %), and lowest among physiotherapists, occupational therapists, psychologists and welfare officers (14 %).

### Questionnaire

Earlier research and theories regarding factors important to older workers and retirement present a complex picture with many facets [39, 40]. To cover most of the known areas of interest, a questionnaire was developed from a literature review and from results of previous studies by the research team regarding different areas important to older workers, retirement and extended working life. The questions in the questionnaire were subdivided into nine theoretical themes. These themes and the number of items in each were: (i) Physical and mental health (9 variables); (ii) Personal finances incentives (7 variables); (iii) Physical work environment (3 variables); (iv) Mental work environment (9 variables); (v) Work pace and working time (9 variables); (vi) Attitude to seniors in the organisation (7 variables); (vii) Possibility for skill development (9 variables); (viii) Motivation and work satisfaction (7 variables); and (ix) Family/leisure pursuits and attitude to retirement in society (5 variables). The majority of the chosen questions in the questionnaire had previously been validated in other studies. A pilot study was performed to ensure that the final questionnaire fulfilled the objectives.

In the analysis, the basis for the ‘self-rated health’ variable was the following variable in the questionnaire: ‘I am currently experiencing good health/well-being’. The response options were dichotomised from four to two variables, i.e. from both highly agrees and agrees to just ‘Highly agree’, and from both partly disagree and disagree to just ‘Disagree’. The variable ‘diagnosed disease’ was based on the variable ‘I have a diagnosed disease’, with the response opinions ‘Yes’ or ‘No’. To take the analysis one step forwards, the variable ‘I have a diagnosed disease which is a hindrance in my daily work’ was also used in the analyses. Those response options were also dichotomised from four to two variables, i.e. from highly agrees and agrees to ‘Highly agree’, and from partly disagree and disagree to ‘Disagree’. Those who did not answer this question were added to those who disagreed, because the question was a follow-up question to the earlier described question ‘I have a diagnosed disease’ (Yes/No). A categorisation of diagnoses was not made in this study. The variable ‘Planned retirement’ was based on a question as to whether the participant believed he/she could work until 55–59, 60–64, 65, or ≥66 years of age. The response options were dichotomised at 65 years of age (i.e., working until <65 versus ≥65 years of age). The reason for doing that, was because 65 years is the most common retirement age in Sweden, and to leave working life before is seen as an early retirement. On the other questions, the participants had five response options ranging from ‘Fully agree’ to ‘Fully disagree’. In the present study, the answers to each variable were sorted into three categories: ‘Disagree’, ‘Partly agree’ and ‘Agree’.

### Statistical analysis

Regarding the four aims of the study, we used the following strategy for the statistical analyses:

#### Relationship between the health ratings

The relationship between the two health ratings (self-rated health and diagnosed disease) was presented in frequencies. The difference between self-rated and diagnosed disease was analysed in cross-tabulation and tested with McNemar’s test. In addition, separate analyses were performed for subgroups based on gender, age, occupation and planned retirement.

#### Relationship between the nine themes and the health ratings: diagnosed disease and self-rated health

This was analysed and tested with logistic regression models (generating odds ratios [OR], 95 % confidence intervals [CI], and *P*-values) to identify statements within the nine themes that were associated with the health outcomes. For each of the outcome variables, we used the following analytical strategy:

##### Analyses within each of the nine themes

First, univariate analyses were carried out; in other words, the relationship was evaluated for one variable at a time, and variables with *P* < 0.05 in the regression analyses were retained. In the second step, the variable with the lowest *P*-value (if *P* < 0.05) was retained and all other statements were tentatively included, one variable at a time. In the third step, the two statements with the lowest *P*-values (if both *P* < 0.05) were retained and the remaining statements were tentatively included one variable at a time. This procedure continued as long as *P* < 0.05 for all variables included.

##### Analyses including all nine themes

The analysis started by including the selected variables from theme (i) and theme (ii) in a multivariate model (i.e., the themes ‘Physical and mental health’ and ‘Personal finances incentives’). Variables with *P* < 0.05 were retained in the model for the next step, which also included the selected variables from theme (iii). This procedure continued until all nine themes were included in a final model. To check once more whether the model was robust, the variables excluded from the nine themes were tested one at a time with the final model.

#### Relationships between the health ratings, the nine themes and planned retirement

Logistic regression analysis was used for evaluating the relationships between the health ratings and to check whether the participant believed he/she could work until <65 versus ≥65 years of age. Both univariate (i.e., one health measure at a time) and multivariate analyses were performed.

## Results

### Relationships with health ratings

The relationship between the two health ratings (self-rated health and diagnosed disease) was tested by a cross tabulation. A total 25 % stated that they have a diagnosed disease and low self-rated health; 8 % stated no diagnoses, but low self-rated health; 20 % stated that they have a diagnosed disease, but a high self-rated health; 48 % stated no diagnoses and a high self-rated health (*P* < 0.001).

In the study population, 53 % thought they could work until 55–64 years of age, and 47 % thought they could work until 65 years of age or beyond (Table 1). Among those with high self-rated health, 62 % believed they could work until 65 years of age. Of those who had a diagnosed disease, 46 % thought they could work until 65 years of age or beyond. Furthermore, 55 % stated that their diagnosed disease was caused by their work, and 62 % stated that their diagnosed disease was an obstacle in their daily work.

**Table 1:** Proportion of respondents with high or low self-rated health and diagnosed disease; their age groups, self-estimated retirement age, gender and occupation

		High self-rated health	Low self-rated health	No diagnosed disease	Diagnosed disease
Median age in the group		58 years	59 years	58 years	59 years
55–59 years of age (*n* = 1060)		69 %	31 %	58 %	42 %
60–64 years of age (*n* = 696)		65 %	35 %	52 %	48 %
Gender^α^	Women (*n* = 1486)	67 %	33 %	56 %	44 %
	Men (*n* = 263)	71 %	29 %	58 %	42 %
Occupation^α^	Physician (*n* = 294)	67 %	33 %	55 %	45 %
	Nurse (*n* = 362)	69 %	31 %	59 %	41 %
	Nursing assistant (*n* = 273)	70 %	30 %	56 %	44 %
	Physiotherapist (*n* = 118)	68 %	32 %	54 %	46 %
	Occupational therapist (*n* = 49)	65 %	38 %	74 %	26 %
	Welfare officer (*n* = 116)	62 %	38 %	62 %	38 %
	Psychologist (*n* = 75)	67 %	33 %	63 %	37 %
	Psychiatric nursing assistant (*n* = 82)	54 %	46 %	45 %	55 %
	Medical secretary (*n* = 342)	69 %	31 %	55 %	45 %
	Non-defined occupation (*n* = 42)	69 %	31 %	38 %	62 %

Men, and those in the age group 55–59 years, had a higher frequency of self-rated health, whereas women and those aged 60–64 years had more diagnosed disease. The occupation with most diagnosed disease was psychiatric nursing assistant, while nursing assistant had the highest self-rated health.

### Relationship between the nine themes and the health ratings

#### Statements associated with low self-rated health

In the final multivariate model, eight statements from five of the nine themes were associated with low self-rated health (Table 2): two statements from each of the themes ‘physical and mental health’, ‘work pace and working time’ and ‘attitude to seniors in the organisation’, and one statement from each of the themes ‘physical work environment’ and ‘motivation and work satisfaction’. The highest ORs were observed for agreement with the statement: ‘I feel physically worn out’ (OR 4.1, 95 % CI 2.8–6.1) and partial agreement with ‘I am satisfied with my present work situation’ (OR 2.9, 95 % CI 1.8–4.4). The OR changed only marginally when the background variables of age, gender, marital status and occupation, were included in the final models to check the risk of possibilities of confounders (data not shown).

**Table 2:** Distribution regarding ‘self-rated health’ outcomes for statements in the final multivariate model. Increased odds ratio (OR) indicates that the individual experienced low self-rated health (95 % confidence intervals (CI); Ref = reference)

Area	Statement	Agree/disagree with the statement	Univariate estimate		Multivariate model	
			OR	95 % Cl	OR	95 % Cl
Physical and mental health	I feel mentally worn out.	Disagree	Ref.		Ref.	
		Partly agree	5.0	3.8–6.6	1.6	1.1–2.3
		Agree	9.0	6.9–12	1.8	1.3–2.6
	I feel physically worn out.	Disagree	Ref.		Ref.	
		Partly agree	5.8	4.4–7.6	2.5	1.8–3.6
		Agree	13	9.2–17	4.1	2.8–6.1
Physical work environment	I can usually manage the physical working demands of my daily work.	Agree	Ref.		Ref.	
		Partly agree	3.2	2.1–4.9	1.9	1.1–3.2
		Disagree	3.1	2.2–4.4	1.7	1.1–2.7
Working pace and working time	I seldom feel rested.	Disagree	Ref.		Ref.	
		Partly agree	3.6	2.7–4.9	1.6	1.1–2.2
		Agree	7.9	6.2–10	1.9	1.3–2.7
	I feel that I get enough rest/relaxation between my working days.	Agree	Ref.		Ref.	
		Partly agree	9.5	7.3–12	2.3	1.6–3.3
		Disagree	4.8	3.6–6.4	2.1	1.5–3.0
Attitude to senior in the organization	The manager’s attitude towards senior employees is positive at my workplace.	Agree	Ref.		Ref.	
		Partly agree	2.5	1.8–3.5	0.7	0.4–1.0
		Disagree	2.5	1.9–3.3	1.4	1.0–2.0
	I feel discriminated against in my workplace because of my age.	Disagree	Ref.		Ref.	
		Partly agree	3.8	2.5–5.6	2.3	1.4–3.9
		Agree	2.8	1.9–4.1	1.6	1.0–2.6
Motivation and work satisfaction	I am satisfied with my present work situation.	Agree	Ref.		Ref.	
		Partly agree	7.8	5.6–11	2.9	1.8–4.4
		Disagree	4.6	3.6–5.9	2.1	1.6–2.9

#### Statements associated with diagnosed disease

In the final multivariate model, four statements from three of the nine themes were associated with diagnosed disease: one variable from the theme ‘physical and mental health’, one from the theme ‘physical work environment’, and two from the theme ‘working pace and working time’ (Table 3). The statements with the highest OR values were: ‘I can usually manage physical working demands of my daily work’ (OR 2.4, 95 % CI 1.7–3.5), and ‘I feel physically worn out’ (OR 2.3, 95 % CI 1.7–3.2). The OR changed only marginally when the background variables of age, gender, marital status and occupation were included in the final model to check the risk of possible confounders (data not shown).

**Table 3:** Distribution regarding ‘diagnosed disease’ outcomes for statements in the final multivariate model. Increased odds ratio (OR) indicates an individual with a diagnosed disease (95 % confidence interval (Cl); Ref = reference)

Themes	Statement	Agree/ disagree with the statement	Univariate estimate		Multivariate model	
			OR	95 % Cl	OR	95 % Cl
Physical and mental health	I feel physically worn out.	Disagree	Ref.		Ref.	
		Partly agree	2.3	1.7–2.9	1.7	1.3–2.3
		Agree	3.6	2.7–4.7	2.3	1.7–3.2
Physical work environment	I can usually manage physical working demands of my daily work.	Agree	Ref.		Ref.	
		Partly agree	2.1	1.9–4.6	2.3	1.5–3.7
		Disagree	3.1	2.2–4.4	2.4	1.7–3.5
Working pace and working time	The pace of work in my daily work is too rapid.	Disagree	Ref.		Ref.	
		Partly agree	0.8	0.7–1.1	0.6	0.5–0.8
		Agree	1.3	1.0–1.6	0.8	0.7–1.1
	I feel that I get enough rest/relaxation between my working days.	Agree	Ref.		Ref.	
		Partly agree	2.4	1.9–3.0	1.7	1.3–2.2
		Disagree	2.1	1.7–2.7	1.8	1.4–2.4

#### Statements associated with diagnosed disease, which is a hindrance in daily work

For the variable ‘I have a diagnosed disease’, there also was a follow-up question: ‘I have a diagnosed disease which is a hindrance in my daily work’. It was therefore interesting to also investigate the relationship of that variable to the nine themes. In the final multivariate model, five statements from three of the nine themes were associated with ‘my diagnosed disease is a hindrance in my daily work’: two statements from the theme ‘physical and mental health’, one from the theme ‘physical work environment’, and two from the theme ‘personal finance incentives’ (Table 4). The statements with the highest OR values in the multivariate model were: ‘I can usually manage physical working demands of my daily work’ (OR 2.7, 95 % CI 1.5–4.7), and ‘I feel mentally worn out’ (OR 2.1, 95 % CI 1.3–3.3). The OR changed only marginally when the background variables age, gender, marital status and occupation were included in the final model to check for possibilities of confounders (data not shown).

**Table 4:** Distribution regarding ‘I have a diagnosed disease which is a hindrance in my daily work’ outcomes for statements in the final multivariate model. Increased odds ratio (OR) indicates an individual with a diagnosed disease (95 % confidence interval (Cl); Ref = reference)

Themes	Statement	Agree/ disagree with the statement	Univariate estimate		Multivariate model	
			OR	95 % Cl	OR	95 % Cl
Physical and mental health	I feel physically worn out.	Disagree	Ref.		Ref.	
		Partly agree	3.2	2.3–4.5	2.0	1.3–3.0
		Agree	2.3	1.5–3.4	1.5	0.9–2.4
	I feel mentally worn out.	Disagree	Ref.		Ref.	
		Partly agree	2.5	1.8–3.3	1.7	1.2–2.4
		Agree	2.3	1.5–3.6	2.1	1.3–3.3
Personal economic incentives	My intention is to work beyond age 65 to get a better pension.	Agree	Ref.		Ref.	
		Partly agree	1.7	1.1–2.5	1.7	1.1–2.6
		Disagree	1.6	1.1–2.3	1.5	1.0–2.2
	I want to work fewer hours even if I do not maintain the same personal economic standards.	Disagree	Ref.		Ref.	
		Partly agree	1.5	1.1–2.2	1.4	1.0–2.0
		Agree	2.3	1.7–3.2	1.9	1.3–2.6
Physical work environment	I can usually manage physical working demands of my daily work.	Agree	Ref.		Ref.	
		Partly agree	1.2	0.7–2.3	1.1	0.6–2.2
		Disagree	3.6	2.1–6.0	2.7	1.5–4.7

### Relationships between the health ratings, statements and planned retirement

A total 52 % (*n* = 919) of the participants believed they could work until 65 years of age or beyond. In univariate models, self-rated health (high vs. low; OR 3.3, 95 % CI 2.7–4.1) and diagnosed disease (yes vs. no; OR 1.7, 95 % CI 1.4–2.1) were associated with whether they thought they could work until 65 years of age or not. When the health ratings were investigated simultaneously, self-rated health (high vs. low; OR 3.2, 95 % CI 2.5–4.0) with no diagnosed disease (no vs. yes; OR 1.1, 95 % CI 0.9–1.3) was significantly associated with whether the participants thought they could work until 65 years of age or not. To take the analysis of the difference one stage further, the variable ‘I have a diagnosed disease which is a hindrance in my daily work’ was inserted. When the health ratings were investigated simultaneously, self-rated health (high vs. low; OR 2.7, 95 % CI 2.1–3.6) and having a diagnosed disease as a hindrance in daily work (high vs. low; OR 1.8, 95 % CI 1.3–2.4) were significantly associated with whether the participants thought they could work until 65 years of age or not, though not having a diagnosed disease was not (no vs. yes; OR 1.3, 95 % CI 0.9–1.8). However, self-rated health was still more highly associated with whether the participants thought they could work until 65 years of age or not.

In the next step of the analysis, the background variables of age, gender, marital status and occupation were included in the final model to check the risk of confounders. Despite this, the OR changed only marginally (data not shown).

## Discussion

This cross-sectional study examined employees’ subjective experienced self-rated health compared with objectively diagnosed disease, and the relationship of these with planned retirement. As a part of the exploration and deriving a theory development on older workers’ situations, a questionnaire grounded in previous literature and regarding nine theoretical themes was used in the study.

High and low self-rated health were stated to be more strongly associated with whether older workers believed they could continue working beyond 65 or not. That was consistent even though diagnosed disease was a hindrance in the older employees’ daily work. Of the nine themes chosen to cover older workers’ lives and work situations, ‘physical and mental health’, ‘physical work environment’ and ‘working pace and working time’ were associated with diagnosed disease. However, these three themes as well as the themes ‘attitude to seniors in the organisation’ and ‘motivation and work satisfaction’ were associated with self-rated health. The results showed that 27–29 % of the respondents gave inconsistent answers regarding self-rated health and diagnosed disease. That showed an important difference between older workers’ own experience, and the effect of their self-rated health and their diagnosed diseases.

Today, diagnosed disease is used to determine the need for economic assistance during sick leave and for a disability pension in the social insurance system in many countries [31–33]. In this study, ‘diagnosed disease as a hindrance in daily work’ was the only health parameter associated with personal economic situation, but was not as highly associated as physical work environment or mental and physical capability. In the present study, those aged 55–59 years reported higher self-rated health and less diagnosed disease than those aged 60–64 years of age. The participants with high self-rated health and no diagnosed disease were most likely to believe they could work until 65 years of age or beyond. The results indicated that self-rated health was a better predictor of extended working life than diagnosed disease. This result is similar to those of earlier studies that stated self-rated health to be a better health predictor than diagnosed disease or disability, retirement and mortality [31–37]. Therefore, these earlier studies and the results from our study seem to indicate that it is not reliable to only determine people’s health with an objective health rating of diagnosed disease for retirement or their potential to extend their working life. However, this study does not have the intention to change the formal system that grants retirement to only include subjective health ratings. The interest is in how we could make working life more sustainable for people. When self-rated health is stated to be an important factor for an extended working life, we think it is important to make interventions that could increase an individual’s self-rated health, as well as reduce their diagnosed disease.

Self-rated health seems to include more information than diagnosed disease on how people experience their total work situation in relation to their health and wellbeing. Both subjective self-rated health and objectively diagnosed disease were mostly associated with older workers’ physical capability, physical work environment, working pace and opportunities to get sufficient rest and relaxation between their working days. In addition, self-rated health was associated with satisfaction in their work situations, the attitude of managers to older workers in the organisation, and whether individuals experienced age discrimination. Previous studies have shown that older people who experience good quality of life and have meaningful tasks experience better health [11, 20–22], while health among individuals with low satisfaction and low occupational grade improves after they leave working life [25]. In the present study, these factors appeared to be particularly associated with self-rated health.

Secondary findings that need to be further investigated are that diagnosed disease and low self-rated health proved to be more common among women than men in this study. Earlier studies have mostly associated women’s preference for early retirement with the retirement of their older husbands [45]. However, men who live with an older woman also plan to retire early, along with their older wife [11]. Therefore, in future studies, the importance of women’s health experience in relation to planned retirement needs to be analysed in depth. Previous studies have also identified educational level, occupational status and working conditions as predictors of health, ageing and ability to participate in working life [10, 26, 46, 47]. Occupational differences therefore also need to be further investigated in a future study. A new study is already planned to analyse specific diseases that might be predictive of early or later retirement.

### Limitations

The questionnaire was formulated from a theoretical model of ageing and themes with importance for work life participation [41–44]. The majority of the statements in the questionnaire have previously been validated in other surveys, but some questions were new and not validated. Another limitation was that although the number of participants in the study was relatively large, a potential weakness was that 36 % of the individuals contacted did not participate. Unfortunately, we were unable to evaluate this dropout rate in depth due to lack of information about the non-respondents. A further limitation of the study was that everyone included in the study population was employed, and we do not know whether people with poor health had already left the workforce. Due to this ‘healthy worker effect’, selection bias must be considered for the study. However, internal comparisons are reported to be one of the most effective ways of reducing the healthy worker effect, and the individuals in this study worked in the same workforce [48]. The respondents in the study were mostly women, and this reflects the gender distribution among people employed in the healthcare and medical sector. We do not regard this as a major shortcoming of the study, since the models were robust even after adjusting for gender, age and marital status. Furthermore, older people were described as rating their self-rated health more highly than younger people did [27, 29]. However, the study population were in the same age group (55–64 years of age), so we do not regard this as a major shortcoming. Finally, the study was a cross-sectional study and only in one working sector. Therefore, a limitation is that we do not have any information on how the participants’ health rates have developed over time, and whether the result is specific for individuals working in health care. Therefore, we will test the result in a new study, following the health development in a cohort with individuals from different occupations aged 55–75 years.

## Conclusion

Self-experienced health is a subjective health rating, and diagnosed disease is an objective one. Self-rated health was more highly associated with older workers’ retirement planning in this study than diagnosed disease was. The most important factors for older workers’ experience of a good self-rated health were their physical and mental fatigue, potential for revitalization, having meaningful activities and occupations that gave work satisfaction, as well as experiencing age discrimination and attitude of managers to them as seniors. Related to findings in this study about older workers’ health, considerations of initiative to extend working life might: i) adjust the work pace to older workers’ capacity and/or provide extra time for rest and relaxation between working sessions; ii) combat age discrimination and engender more positive attitudes towards older workers among managers and within organisations; and iii) increase motivation and satisfaction through interesting tasks and occupations. We will, in a forthcoming intervention study, investigate if such initiatives could be a sustainable way to improve older workers’ health and possibly increase the number of work hours they can and want to contribute to the national economy.
